# Sex differences in autonomic functions and cognitive performance during cold-air exposure and cold-water partial immersion

**DOI:** 10.3389/fphys.2024.1463784

**Published:** 2024-10-16

**Authors:** Youngsun Kong, Md Billal Hossain, Riley McNaboe, Hugo F. Posada-Quintero, Matthew Daley, Krystina Diaz, Ki H. Chon, Jeffrey Bolkhovsky

**Affiliations:** ^1^ Biomedical Engineering, University of Connecticut, Storrs, CT, United States; ^2^ Analog Devices, Wilmington, MA, United States; ^3^ Naval Submarine Medical Research Laboratory, Groton, CT, United States; ^4^ Leidos, Reston, VA, United States

**Keywords:** sex differences, cold, autonomic functions, cognitive performance, electrodermal activity, heart rate variability, memory, reaction time

## Abstract

**Introduction:**

This study investigated the differences between males and females in autonomic functions and cognitive performance during cold-air exposure and cold-water partial-immersion compared to a room temperature-air environment. Although several studies have investigated the effects of cold-air or cold-water exposures on autonomic function and cognitive performance, biological sex differences are often under-researched.

**Methods:**

Twenty-two males and nineteen females participated in the current study. Subjects completed a battery of cognitive tasks based upon those used within the Defense Automated Neurobehavioral Assessment (DANA), consisting of five subtasks that assess simple and procedural reaction time, spatial manipulation, attention, and immediate memory. In total, subjects took the battery within a 15-minute period across 30-minute intervals throughout the duration of environmental exposure. Across three separate days, subjects were exposed to three different environmental conditions: room temperature air (23°C), cold air (10°C), and cold water (15°C; in which subjects were immersed up to their necks). Room temperature and cold-air conditions consisted of five sessions (about 2.5 h), and the cold-water condition consisted of three sessions (about 1.5 h). During each experimental condition, physiological data were collected to assess autonomic function, including electrodermal activity (EDA) data and heart rate variability (HRV) derived from electrocardiogram signals.

**Results:**

Females showed slower reaction time in spatial manipulation tasks, immediate memory, and attention during cold-air exposures compared to room temperature air, whereas the performance of males were similar or better during cold-air exposures compared to room temperature air. Cold-water immersion affected the immediate memory performance of males. Both males and females exhibited smaller EDA amplitudes during cold-air and cold-water conditions compared to room temperature air. For HRV, only male subjects exhibited significantly greater values in low-frequency and very-low-frequency components during cold air exposure compared to the normal condition.

**Discussion:**

Sex introduces important differences in cognitive performance and autonomic functions during exposure to cold-air and cold-water. Therefore, sex should be considered when assessing the autonomic nervous system in cold environments and when establishing optimal thermal clothing for performance in operational environments. Our findings can assist with determination of operational clothing, temperature in operating environment, and personnel deployment to operational sites, particularly in settings involving both males and females.

## Introduction

Prolonged exposure to cold temperatures can have negative effects on cognitive performance such as attention, mental processing speed, executive function, and memory (Pilcher et al., 2002; Falla et al., 2021; Yang et al., 2021). These effects are likely associated with the body’s natural physiological and thermoregulatory responses. When humans are exposed to cold air or immersed in cold water, their body undergoes thermoregulatory responses to maintain their core temperature within a narrow range (Osilla et al., 2018). Additionally, immersion in cold water is influenced by an elevated hydrostatic pressure due to the density of water, which is 800 times higher than that of air (Jimenez et al., 2010). The sympathetic nervous system (SNS) activates vasoconstriction in the peripheral blood vessels, reducing blood flow to the skin and other superficial tissues, which decreases overall heat loss (Campbell, 2008). The SNS also activates brown adipose tissue capable of converting energy into heat (Lee et al., 2013). Studies have also shown increases in parasympathetic nervous system (PNS) activity after initial SNS response, and also during cold habituation (Harinath et al., 2005; Mäkinen et al., 2008; Castellani and Young, 2016; Lundell et al., 2020). The mechanisms and interplay of SNS and PNS functions employed during thermoregulation are unclear; assessing the physiological signals of the autonomic nervous system (ANS) may help clarify these mechanisms and their associations with cognitive performance in cold-air exposures and cold-water immersion.

The ANS is often assessed via heart rate variability (HRV), which is typically derived from data collected through an electrocardiogram (ECG), a non-invasive measure of the electrical activity generated by the heart (Shaffer and Ginsberg, 2017). HRV can provide insights into the interplay of cardiac SNS and PNS functions during cold exposure, although it is often difficult to distinguish the exact mechanism behind each function/system. Using HRV analysis, researchers found a significant increase in PNS activity from subjects who underwent cold-air exposure or cold-water immersion (Mäkinen et al., 2008; Lundell et al., 2020). Despite their insightful outcomes, these studies were limited to assessing cardiac PNS, as none of the HRV indices provided exclusive assessment of cardiac SNS. For example, the high frequency component of HRV is primarily influenced by cardiac PNS, whereas both cardiac SNS and PNS have been found to affect the low frequency components of HRV (Shaffer and Ginsberg, 2017).

Electrodermal activity (EDA) has shown promising ability to exclusively assess SNS activity (Posada-Quintero and Chon, 2020). EDA is a non-invasive measure of sudomotor activity, which is activated by the sympathetic function. EDA devices measure the variation in the electrical conductance of the skin predominantly affected by the SNS (Shields et al., 1987). This advantage enables researchers to get insights into the ANS, with more reliable assessments of SNS activity using EDA alone or in complement with HRV (Visnovcova et al., 2013; Posada-Quintero and Bolkhovsky, 2019; Ghiasi et al., 2020). EDA is often decomposed into two distinct components: phasic and tonic. The phasic component reflects the rapid EDA changes in response to a startle-like stimulus or event, while the tonic component reflects the overall level of electrical conductance of the skin, influenced by factors such as baseline arousal, hydration levels, and skin temperature (Posada-Quintero and Chon, 2020). Additionally, the time-and-frequency-domain analysis, such as the time-varying index of EDA (TVSymp), has shown high sensitivities in assessing SNS activity (Posada-Quintero et al., 2016; Kong et al., 2021b; Tran et al., 2022; Kong et al., 2023).

Although the effects of cold air exposure or cold-water immersion on autonomic function and cognitive performance have been studied, many of these studies have not considered biological sex as a confounding factor. Sex is an important factor in both the thermoregulation mechanism and the assessment of autonomic function (Tikuisis et al., 2000; Kaikaew et al., 2018; Mengel et al., 2020). Males and females generally have different body compositions that can affect thermoregulation, such as body fat distribution, muscle mass, hormonal differences, and body size (Kaciuba-Uscilko and Grucza, 2001; Castellani and Young, 2016; Bredella, 2017). Furthermore, a meta-analysis by Koenig and Thayer concluded that sex differences in HRV should be taken into account in HRV analysis (Koenig and Thayer, 2016). Studies have shown that EDA has different dynamics between males and females (Bari, 2020; Kong et al., 2021a), but these studies did not exhibit statistically significant differences in EDA metrics. Given that males and females can react to cold exposure differently, we hypothesized that there are sex differences in both EDA and HRV metrics and cognitive performance during cold environments. The current study investigated differences in cognitive function and autonomic function during cold-air exposure and cold-water immersion of males and females. Understanding sex differences in cold environments can be beneficial for operational conditions where both males and females work, such as military settings.

## Materials and methods

### Subjects

Data were collected from 41 subjects, whose mean age was 24.32 ± 5.03 years old. Subjects were recruited from around the University of Connecticut (UConn). Subjects were asked to come to the experiment site for three different days, during which they performed a cognitive task battery while exposed to one of three different environmental conditions: room temperature air (i.e., “normal”) (23°C), cold-air (10°C), and cold-water (15°C). Subjects were compensated $50 (USD) a day and given an extra $150 (USD) if they completed all 3 days/conditions. Subjects underwent an informed consent procedure and completed a questionnaire of demographic information prior to participating in the experiment.

### Stimuli and materials

#### Task battery for the assessment of cognitive performance

Used within the experimental cognitive task battery were five tasks derived from the Defense Automated Neurobehavioral Assessment (DANA), a battery designed to evaluate cognitive and psychological performance in operational environments (Lathan et al., 2013). This included 1) go/no-go (GNG), 2) spatial discrimination (SPD), 3) procedural reaction time (PRO), 4) simple reaction time (SRT), and 5) code substitution (CDS). This set of cognitive task battery evaluates abilities ranging from simple reaction time (SRT) and executive functioning with easy decision-making capabilities (PRO) to measure of sustained attention and impulsivity (GNG), visuospatial analytic ability (SPD), and short-term memory (CDS) (Lathan et al., 2013). Note that DANA has shown consistency and reliability across simulated environmental conditions, including a cold condition (around 3°C) (Haran et al., 2015). The battery was administered on a Galaxy Tab S7 tablet device (Samsung, S. Korea), using the Inquisit Millisecond software (Millisecond Software, LLC). Supplementary Figure S1 shows an example of screenshots of the task battery.

#### Go/No-go (GNG)

Subjects were shown a rectangle on the screen in either a horizontal or vertical orientation. They were then instructed to press the space bar button as fast as possible if the rectangle turned green, indicating a “go” signal, regardless of its orientation. They were also told not to respond if the rectangle turned blue, indicating a “no-go” signal. The goal of this task, which took approximately 2 min and 30 s to complete, was to assess subjects’ speed and accuracy of selecting targets, including their ability to make commissions and omissions. Subjects’ average reaction time and the number of incorrect responses were used to evaluate their performance.

#### Spatial discrimination (SPD)

Subjects were shown a set of two histograms, each consisting of 4 bars, on the screen. After the initial presentation of the first set, a second set of histograms was shown, which were rotated by either 0°, 90°, 180°, or 270° angles. The subjects were then required to determine whether the two sets of histograms were congruent or not. The purpose of this task was to assess the subjects’ spatial manipulation abilities, and it took approximately 3 min to complete. Subjects’ performance was measured by calculating their average reaction time and the number of incorrect responses.

#### Procedural reaction time (PRO)

Subjects were shown a horizontal row of four boxes onscreen. When a box turned red, subjects were asked to tap on buttons presented at the bottom of the screen that corresponded to each of those boxes. The objective of this task was to assess the subjects’ reaction time and their ability to accurately respond while inhibiting premature responses. The task took approximately 90 s to complete, depending on the individual’s reaction time. The subjects’ average reaction time, the number of incorrect responses, and the number of premature responses were calculated to evaluate their performance.

#### Simple reaction time (SRT)

Subjects were instructed to tap anywhere onscreen as soon as a red circle appeared. The task took approximately 90 s to complete and was designed to assess the subjects’ reaction time. The subjects’ average reaction time and the number of lapses (defined as instances where the reaction time exceeded 0.5 s) were calculated to evaluate the subjects’ ability to sustain attention and quickly respond to stimuli.

#### Code substitution (CDS)

Subjects were shown a matrix of symbols and were instructed to enter the corresponding digits as quickly and accurately as possible, based on a key of symbol-digit pairs. The matrix of symbols was randomized for each trial. Subjects were given 2 min to complete as much of the matrix as they could. The purpose of this task was to evaluate the subjects’ immediate memory and attention abilities. Subject performance was evaluated by calculating the average reaction time of each input and the error rate, which was determined by dividing the number of incorrect responses by the total number of responses.

### Experimental conditions

The experiment assessed cognitive performance and physiological response in three different environmental conditions: 1) the “normal-air condition” which had a room temperature of 23°C, 2) the “cold-air condition” which had a room temperature of 10°C, and 3) the “cold-water condition” which required subjects to be immersed up to the neck in water with a temperature of 15°C. The relative humidity was maintained at 35% ± 1% for the normal-air condition; while it was 57% ± 1% for the cold-air condition. The subjects experienced each condition on three different days at a similar time, with the order of the environmental conditions pseudo-randomly assigned. To control for clothing insulation across subjects, subjects were provided with clothing to wear in each condition. Clothing was worn over all physiological sensors. For the normal-air and cold-air conditions, subjects were given a long sleeve cotton shirt, long cotton pants, and cotton ankle socks, which provided a clothing insulation (Clo) rating slightly less than 1, in which 1 Clo = 0.155 m^2^K/W. To perform the task battery, subjects used a smart tablet while seated at a desk. For the cold-water condition, subjects wore a 3 mm Henderson Aqua Lock dive wetsuit and 3 mm Aqua Lung diving boots (approximately 0.25 Clo) (Tikuisis and Keefe, 1996). During the cold-water sessions, subjects sat in water filled up to the neck, in the Whitehall S110M Mobile Whirlpool bathtub, and a bathtub tray was provided for the subjects to use while performing the tablet-based cognitive task battery.

### Mean body temperature

Skin temperature was calculated from three different body regions. Dermal patch sensors (VitalSense, Philips Respironics, Bend, Oregon, United States) were used to collect the skin temperature data from the back of the dominant hand, the left calf, and the forehead. Also, subjects’ core temperature was obtained using an ingestible capsule sensor (VitalSense, Philips Respironics, Bend, Oregon, United States) (Travers et al., 2016). These patches and ingestible sensors transmitted data to the Equivital EQ02+ LifeMonitor’s Sensor Electronic Modules (Equivital, Cambridge, United Kingdom) every 5 seconds. Using ISO Standard weighting coefficients (ISO 9886:2004 en, 2004), the area-weighted skin temperature (T_skin_) was calculated from the three skin temperature measurements. Since only T_skin_ data were collected from three locations, the weighting coefficients were proportionally adjusted to have a sum of 1 (Supplementary Table S1). Finally, the mean body temperature (MBT) was calculated using Burton’s formula based on T_skin_ and T_core_ (Burton, 1935; Lenhardt and Sessler, 2006), as follows:

$$T_{skin}=0.21875\times T_{Forehead}+0.15625\times T_{hand}+0.625\times T_{calf}$$

$$T_{MBT}=0.36\times T_{skin}+0.64\times T_{core}$$

### Electrodermal activity (EDA) and heart rate variability (HRV)

Raw EDA signals were recorded at a frequency of 4 Hz using the Empatica E4 (Empatica, Boston, MA, United States), which was placed on the non-dominant wrist. Ag/AgCl electrodes were attached to the index and middle fingers and connected to Empatica E4 to record EDA signals. The raw EDA signals were then decomposed into phasic (PhEDA) and tonic (TonEDA) components using the cvxEDA technique (Greco et al., 2015). PhEDA and TonEDA correspond to fast and slow components of EDA, respectively. The time-varying index of sympathetic activity (TVSymp) was derived from the raw EDA signals. TVSymp is derived from reconstructed signals using components within a frequency range of 0.08–0.24 Hz, and has shown high sensitivity to SNS activity, such as noxious stimuli, stress, and dental pain (Posada-Quintero et al., 2016; Posada-Quintero et al., 2020; Kong et al., 2021c; Kong et al., 2023; Tran et al., 2022). Non-specific skin conductance responses (NSSCR) were calculated by counting the number of skin conductance responses that met a set threshold in a given time period. The threshold is normally set to 0.05 (Posada-Quintero and Chon, 2020). However, three different threshold values were assessed, which are 0.05, 0.01, and 0.005 μS, as cold acclimatization can reduce perspiration rates (Mcmullen et al., 2013).

To obtain HRV, lead-2 electrocardiogram (ECG) signals were recorded using multiple devices, to minimize the effect of data corruption. For normal and cold-air conditions, the ScottCare Chroma Holter Monitor (ScottCare, Cleveland, OH, United States) was used with Ag/AgCl electrodes, which were attached to the upper chest and the left and right lower rib areas. For the cold-water condition, Rozinn RZ153+ Holter Recorder (Cardiac Direct, Ventura, CA) was used with reusable hydrophobic electrodes comprised of carbon black powder and polydimethylsiloxane (CB/PDMS), which were attached to the upper chest and the left lower rib areas (Reyes et al., 2014). Also, ECG signals were collected using the Equivital EQ02+ LifeMonitor for all conditions. Finally, photoplethysmogram (PPG) signals were collected from the non-dominant wrist using Empatica E4.

HRV was calculated from R-peak intervals obtained using an approach based on the complete ensemble empirical mode decomposition (CEEMD) (Torres et al., 2011; Hossain et al., 2019). If R peaks were not identifiable due to artifacts, HRV was calculated using PPG signals (provided they were devoid of noise artifacts). Pulse peaks of PPG signals were obtained using an accurate peak detection technique only when the PPG quality was sufficiently high (Han et al., 2022). The following HRV indices were calculated: high- (HF), low- (LF), and very-low-frequency (vLF) power, LF/HF ratio, heart rate (HR), the root mean square of successive differences (RMSSD) between normal heartbeats, and the standard deviation of the interbeat intervals of normal sinus beats (SDNN).

### Protocol

Subjects were instructed to get a minimum of 8 h of sleep the night before each testing day. They were also asked to avoid consuming any depressants and stimulants, such as nicotine, alcohol, and caffeine, for at least 24-h before a testing session. Finally, subjects were instructed to consume food no less than 3 h before each testing day. All condition testing was conducted at similar times of day on the fourth floor of the Engineering and Science Building at UConn Storrs Campus. On the first day of participation subjects completed a demographic questionnaire and provided information including age, sex, height (in), weight (lbs), waist circumferences (cm), smoking, and fitness (rated on a scale from 1–3: sedentary, moderately, vigorously active); skinfold measurements (mm) were also taken (Jackson and Pollock, 1985). Note that the units for height and weight were then converted to centimeters and kilograms. Different body areas were measured between male and female subjects for skinfold measurements. Skinfold measurements of the chest, abdomen, and thigh were obtained for male subjects, whereas thigh, triceps, and suprailium measurements were collected from female subjects (Jackson and Pollock, 1978; Jackson and Pollock, 1985; Jackson et al., 1980). Body mass index (BMI) was calculated using height and weight. Also body fat was estimated for both male and female subjects using Jackson and Pollock’s 3-site skinfold equations and Siri’s formula (Siri, 1956; Jackson and Pollock, 1978; Jackson et al., 1980).

Subjects were provided clothing, a capsule sensor with a cup of water, three dermal patch sensors, and the Equivital EQ02+ LifeMonitor vest. Before the sensors were placed, the skin areas were cleaned with 70% isopropyl alcohol. They were then asked to attach all electrodes to their respective, aforementioned placements for EDA and ECG recording. They were asked to wear the Empatica E4 on their non-dominant wrist. For the cold-water condition, subjects wore a nitrile glove on the non-dominant forearm to protect the Empatica E4 sensors and electrodes from water.

**FIGURE 1:**
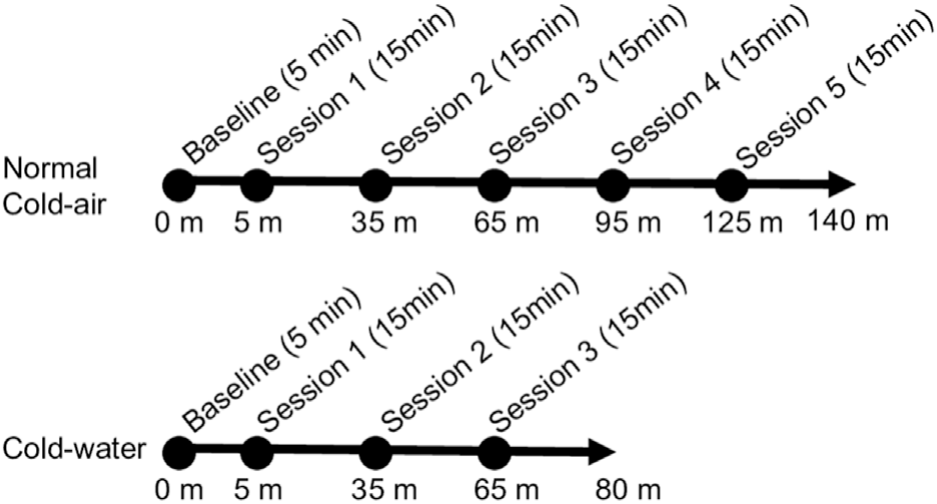
Experimental protocol for the three experimental conditions.

Figure 1 shows the sequence of all environmental conditions. At the start of each condition, 5 min were provided to participants for hemodynamic stabilization before starting the battery of cognitive tasks. Subjects completed two practice sessions of the task battery to minimize the effect of learning on the first day, because a mild learning effect has been observed during the first and second trials of DANA from which our cognitive task battery was derived (Lathan et al., 2013). A total of five experimental sessions of the cognitive task battery were conducted for the normal and cold-air conditions, whereas the cold-water condition consisted of three sessions to minimize the risk of hypothermia. Each session of the battery took approximately 15 min, and the sessions were repeated every 30 min. During each session for the cold-water condition, subjects were immersed in water up to the upper chest to perform the battery of cognitive tasks and up to the neck when not required to conduct the tasks. This research complied with tenets of the Declaration of Helsinki and was approved by the Institutional Review Board at the University of Connecticut (UConn) and the Naval Submarine Medical Research Laboratory (NSMRL).

### Statistics

For EDA and HRV indices, 5-min segments were used from the middle portion of each session (2.5 min forwards and backwards from the midpoint). For EDA indices, mean values for each index were used. R’s LMER function was used to perform a three-way analysis of variance (ANOVA) with a random intercept for participant (Bates, 2005; Bates et al., 2015) to see if there was a significant effect in sex, between conditions and across exposure time, on cognitive task battery performance and physiological metrics. Note that the LMER function treats missing data as missing at random (MAR) by removing rows containing missing responses and by fitting models based on maximum likelihood estimation (De Boeck et al., 2011). If a significant effect was observed, estimated marginal means (i.e., least square means) of the fitted LMER models were obtained, using R’s emmeans function with the Tukey method to perform a pairwise *post hoc* test (Lenth et al., 2019). To compare the normal and cold-air conditions, segments from the five sessions during the task battery were compared (i.e., five-session analysis). To compare the cold-water condition with the other conditions, segments from the first three sessions of the task battery were analyzed (i.e., three-session analysis). For comparisons of demographic data, a *t*-test, chi-square test, and Mann-Whitney U test were used accordingly to see if there were significant demographic differences between male and female subjects.

## Results

### Demographic characteristics

A total of 41 subjects was recruited, including 22 male (aged 25.7 ± 5.9) and 19 female subjects (aged 22.7 ± 3.3). Among them, a male subject participated in sessions involving the cold-water condition and withdrew from participation in the other conditions. The inter-condition interval was 4.3 ± 9.3 days. There was no missing EDA data, whereas a portion of temperature and HRV data were missing due to sensor malfunctions. For the cognitive task, SPD and CDS had missing data due to software errors. Supplementary Table S2 summarizes the number of available data segments between male and female subjects.

Table 1 shows demographic information between male and female subjects. The male and female subjects did not have detectable significant differences in age, BMI, smoking, and exercise frequency. However, there were significant differences in height, weight, waist circumferences, estimated body fat, and thigh skinfolds. Note that these differences have been commonly observed (Durnin and Womersley, 1974; Flegal et al., 2009; Fryar et al., 2018). Supplementary Table S3; Supplementary Figure S2 shows the distribution of BMI and estimated body fat differences between male and female subjects.

**TABLE 1:** Demographic information (Mean ± S.D., except for Smoking with the number of subjects).

	Male	Female	P-values	Statistic method
Age (year)	25.7 ± 5.9	22.7 ± 3.3	0.060	t-test
BMI (kg/m^2^)	24.5 ± 3.3	23.0 ± 3.9	0.195	
Height (cm)**	176.0 ± 8.1	164.1 ± 10.9	<0.001	
Weight (kg)*	75.9 ± 12.0	62.1 ± 14.0	0.002	
Waist (cm)*	87.9 ± 11.9	78.9 ± 13.4	0.028	
Body Fat (%)**	19.8 ± 7.4	28.3 ± 7.8	<0.001	
Thigh Skinfold (mm)*	25.3 ± 15.4	35.8 ± 14.2	0.029	
Smoking (N_subject_)	3	0	0.284	Chi-square test
Exercise Frequency	1.9 ± 0.6	2 ± 0.6	0.315	Mann-Whitney U test

Exercise Frequency (1: sedentary, 2: normal, 3: vigorous). * and ** indicate significant difference between male and female subjects (*p* < .05 and *p* < .001, respectively).

### Sex difference of mean body temperature, autonomic functions, and cognitive performance

Tables 2, 3 summarize our statistical analyses of male and female differences in five-session (normal vs. cold-air) and three-session (all conditions) comparisons, respectively. Note that five-session analyses compare normal and cold-air conditions, while three-session analyses compare cold-water condition with other conditions in the result and discussion sections.

**TABLE 2:** Statistical analysis of male and female differences (normal and cold-air conditions, 5 sessions).

		ANOVA Factor: Sex		ANOVA Factor: Sex and Condition		Post-hoc Analysis (M vs. F for each condition)	
		F	*p*	F	*p*	*p* Normal	*p* Cold-air
MBT*		0.120	0.732	4.180	**0.042**	0.944	0.630
Reaction time of each task	GNG	0.416	0.523	1.809	0.180	N/A	N/A
	SPD	0.031	0.861	32.329	**<0.001**	0.676	0.863
	PRO*	3*10^-4^	0.987	9.057	**0.003**	0.954	0.961
	SRT	0.132	0.718	0.617	0.433	N/A	N/A
	CDS	5.620	**0.026**	18.196	**<0.001**	**0.021**	0.425
Error-related index of each task	GNG IT	0.617	0.437	1.121	0.290	N/A	N/A
	SPD IT	2.707	0.110	0.114	0.736	N/A	N/A
	PRO IT	0.199	0.658	3.532	0.061	N/A	N/A
	PRO FS	1.015	0.320	2.079	0.150	N/A	N/A
	SRT Lap	0.043	0.837	0.036	0.850	N/A	N/A
	CDS Error	0.140	0.711	1.490	0.223	N/A	N/A
EDA	PhEDA	1.128	0.295	12.612	**<0.001**	0.105	0.984
	TonEDA	0.016	0.900	31.079	**<0.001**	0.749	0.599
	TVSymp	1.487	0.230	9.313	**0.002**	**0.048**	0.937
	NSSCR_0.05_	0.767	0.387	20.568	**<0.001**	0.107	0.907
	NSSCR_0.01_	0.448	0.507	18.319	**<0.001**	0.203	0.885
	NSSCR_0.005_	0.520	0.475	11.990	**<0.001**	0.272	0.965
HRV	HR	1.041	0.314	0.095	0.759	N/A	N/A
	HF	0.013	0.908	1.626	0.203	N/A	N/A
	LF	1.230	0.274	7.957	**0.005**	0.985	0.290
	LF/HF	2.671	0.110	0.037	0.848	N/A	N/A
	vLF	2.495	0.122	18.211	**0.033**	0.982	0.050
	RMSSD	0.019	0.891	0.264	0.607	N/A	N/A
	SDNN	0.344	0.561	11.473	**<0.001**	0.999	0.590

The bold font indicates *p*-value < 0.05.

*Significant effects of combined factors of sex, session, and condition between normal and cold conditions [*F*(4,342) = 3.189, *p* = .014].

**TABLE 3:** Statistical analysis of male and female differences (All conditions, 3 sessions).

		ANOVA Factor: Sex		ANOVA Factor: Sex and Condition		Post-hoc Analysis (M vs. F for each condition)
		F	*p*	F	*p*	*p* Cold-water
MBT		0.040	0.843	2.359	0.098	N/A
Reaction time of each task	GNG	0.017	0.896	0.714	0.491	N/A
	SPD	0.084	0.773	9.788	**<0.001**	0.999
	PRO	0.003	0.954	2.647	0.073	N/A
	SRT	0.108	0.744	0.164	0.849	N/A
	CDS	4.897	**0.036**	7.400	**<0.001**	**0.039**
Error-related index of each task	GNG IT	0.228	0.636	0.050	0.951	N/A
	SPD IT	1.106	0.301	0.567	0.568	N/A
	PRO IT	0.141	0.709	1.521	0.220	N/A
	PRO FS	0.225	0.638	0.621	0.538	N/A
	SRT Lap	0.062	0.804	0.170	0.844	N/A
	CDS Error	1.455	0.240	2.669	0.072	N/A
EDA	PhEDA	0.426	0.518	5.519	**0.004**	1.000
	TonEDA	0.005	0.944	8.317	**<0.001**	0.999
	TVSymp	0.189	0.666	4.014	**0.019**	0.999
	NSSCR_0.05_	0.402	0.530	7.191	**<0.001**	0.999
	NSSCR_0.01_	0.225	0.638	5.274	**0.006**	0.984
	NSSCR_0.005_	0.621	0.435	4.363	**0.014**	0.762
HRV	HR	2.073	0.158	1.529	0.218	N/A
	HF	0.053	0.820	1.492	0.227	N/A
	LF	1.133	0.294	0.275	0.759	N/A
	LF/HF	5.370	**0.026**	0.161	0.851	N/A
	vLF	2.052	0.160	1.270	0.282	N/A
	RMSSD	0.001	0.977	0.084	0.919	N/A
	SDNN	0.056	0.814	1.117	0.329	N/A

The bold font indicates *p*-value < 0.05.

### Mean body temperature

Both cold-air and cold-water conditions affected physiological changes to both males and females. Both male and female subjects showed significantly higher MBT in the normal condition compared to the cold-air condition (5 sessions, *p* < .001) (Supplementary Figure S3).

However, no statistical difference in MBT was observed between males and females. Note that core temperature showed no significant effect of sex or combined factors of sex and condition *per se*, although it was marginally greater in females compared to males in the three-session analysis [*F* (1,36) = 3.824, *p* = 0.058].

### Cognitive performance

**FIGURE 2:**
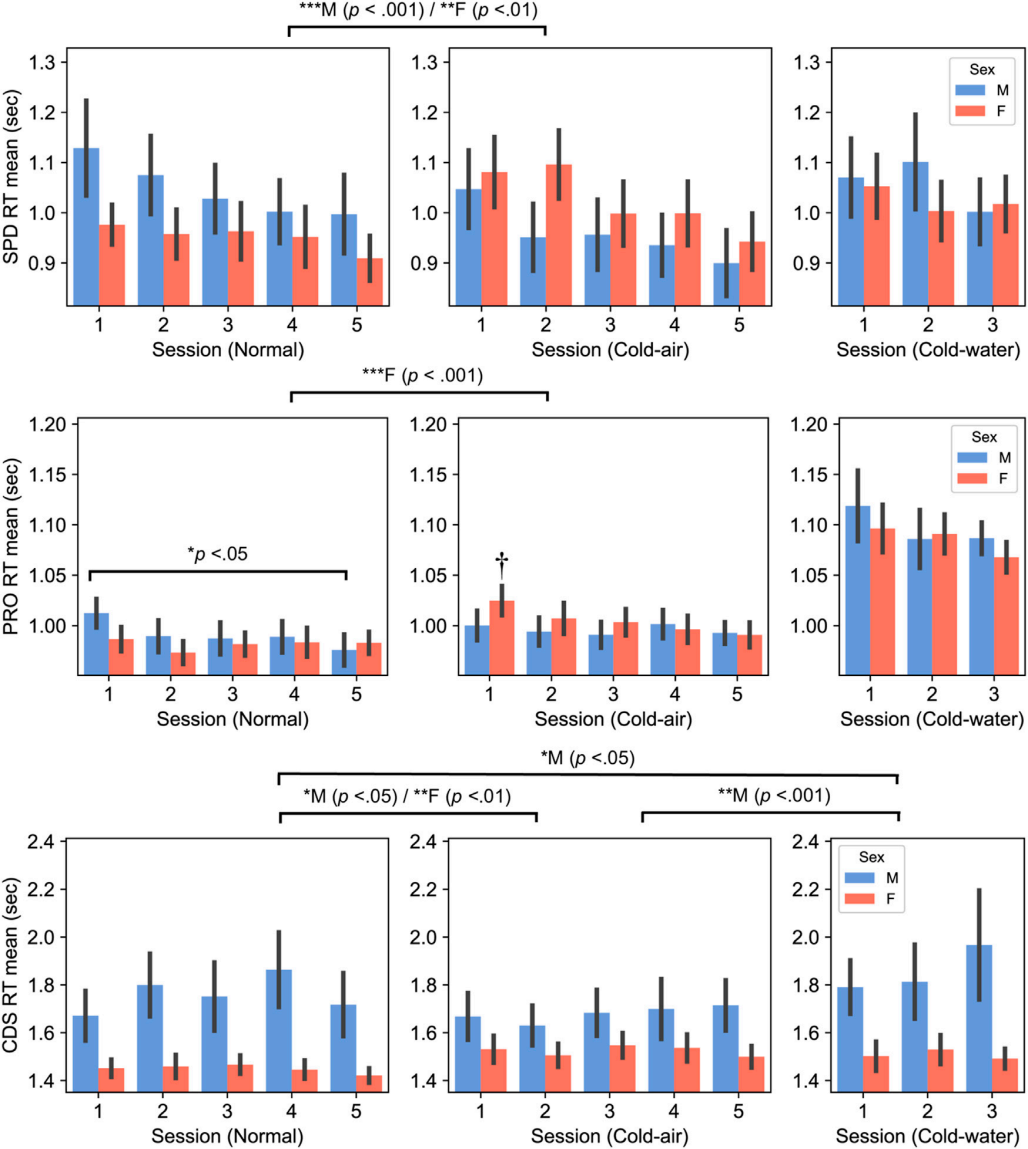
Spatial discrimination (SPD) (top panel), Procedural reaction time (PRO) (middle panel), and Code Substitution (CDS) (bottom panel), RT between males and females (Mean ± SEM). *P*-values displayed at the top of the figure indicate significant differences between the normal and cold-air/water conditions for each sex. † indicates significant difference with normal 1–5 sessions of female subjects (*p* < 0.001 for session 2 and *p* < 0.05 for others).

Cognitive performance exhibited distinct patterns between males and females (Figure 2). Females showed significantly slower reaction times in the cold air-condition compared to the normal condition for SPD (*p* = .002) and for PRO (*p* < .001), whereas males showed significantly faster reaction times of SPD in the cold-air condition (compared to the normal condition, *p* < .001).

Interestingly, male subjects’ PRO reaction time significantly decreased in session 5 compared to session 1 (*p* < .05) in the normal condition, while female subjects showed significantly slower reaction time in the first session in the cold-air condition compared to all the other sessions in the normal condition (*p* < .05). The former is likely due to a learning effect, despite the inclusion of the practice sessions, and the latter may indicate that cold environment affects performance of females during the initial exposure, while the combination of learning effect and acclimation in the cold environment mitigates the affected performance.

Similarly, CDS reaction time exhibited opposite patterns between males and females. Female subjects exhibited significantly slower reaction time in the cold-air condition compared to the normal condition (five-session analysis, *p* = .008). Conversely, male subjects showed significantly faster reaction time in the cold-air condition compared to the normal condition (five-session analysis, *p* = .022). Additionally, male subjects showed a significantly increased reaction time in the cold-water condition compared to the normal (*p* < .05) and cold-air conditions (*p* < .001). Nevertheless, female subjects showed significantly faster reaction time than male subjects in normal condition in five-session analysis (*p* = .021) and in cold-water condition in three-session analysis (*p* = .039).

### Electrodermal activity (EDA)

**FIGURE 3:**
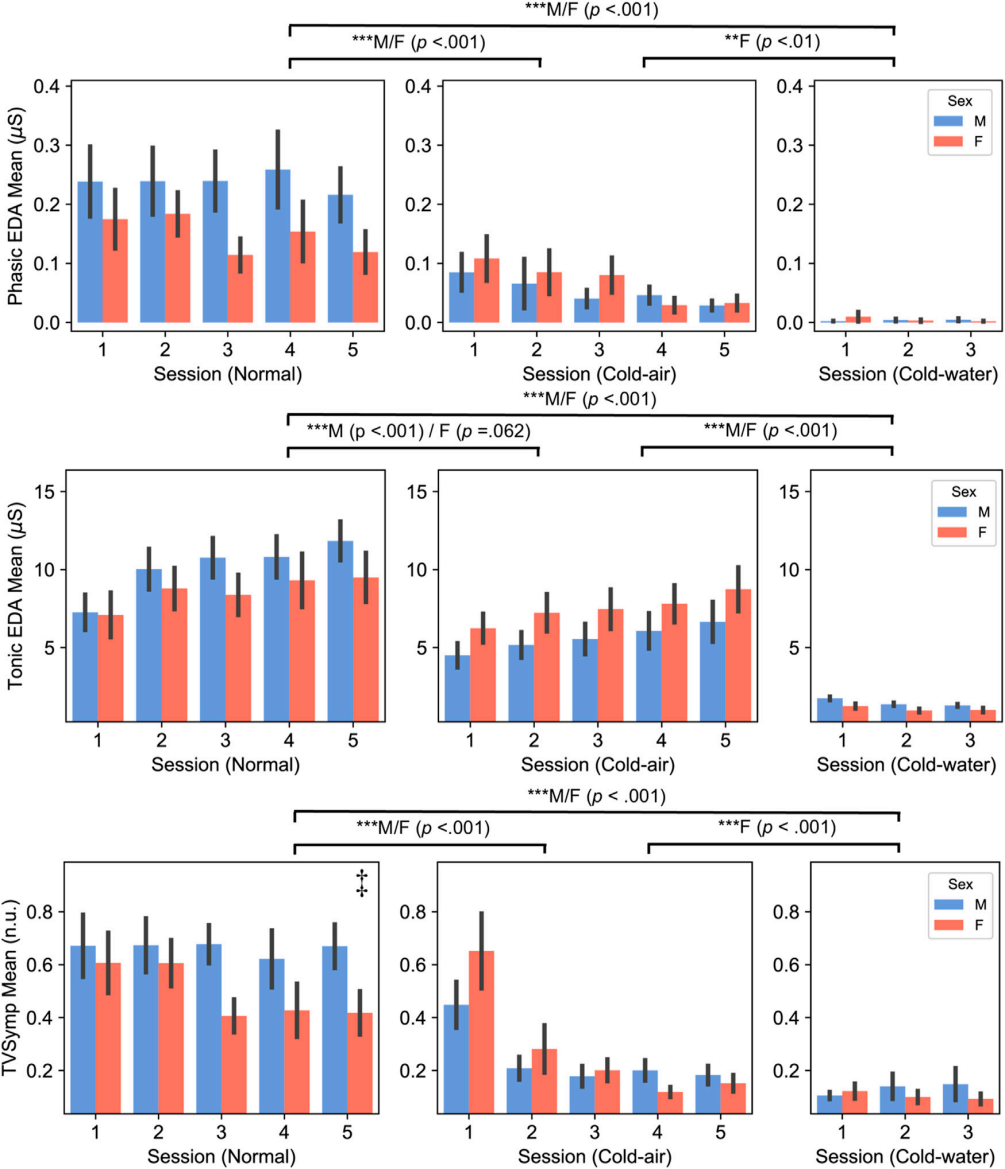
Phasic (top panel) and Tonic (middle panel) components of EDA, and TVSymp (bottom panel) between male and female subjects (Mean ± SEM). *P*-values displayed at the top of the figure indicate significant differences between the normal and cold-air/water conditions for each sex. ‡ indicates there was significant difference between males and females in the normal condition.

All EDA indices showed similar patterns between males and females, with decreased amplitudes during cold-air and cold-water conditions compared to the normal condition (Figure 3; Supplementary Figure S4). Both male and female subjects showed significantly higher PhEDA,TonEDA, and TVSymp in the normal condition compared to the water condition (*p* < .001). Additionally, both male and female subjects showed significantly higher PhEDA and TVSymp in the normal condition compared to the cold condition (*p* < .001). Both male and female subjects showed significantly lower TonEDA in cold water compared to the other conditions (*p* < .001). For NSSCR with all thresholds, both male and female subjects showed significantly higher values in the normal condition than in the other conditions (*p* < .001), and also higher values in the cold-air condition than in the cold-water condition (*p* < .01).

On the other hand, PhEDA, TonEDA, and TVSymp also showed different patterns between males and females. In three-session analysis, only female subjects showed significantly lower PhEDA and TVSymp in the cold-water condition compared to cold-air condition (*p* < .01), whereas males did not. Additionally, only male subjects showed a significantly lower TonEDA in the cold-air condition compared to the normal condition (*p* < .001), whereas females did not. Interestingly, a significant difference in TVSymp was found between male and female subjects in the normal condition (five-session analysis, *p* = .048). Sex did not have a significant effect on the NSSCR features.

### Heart rate variability (HRV)

**FIGURE 4:**
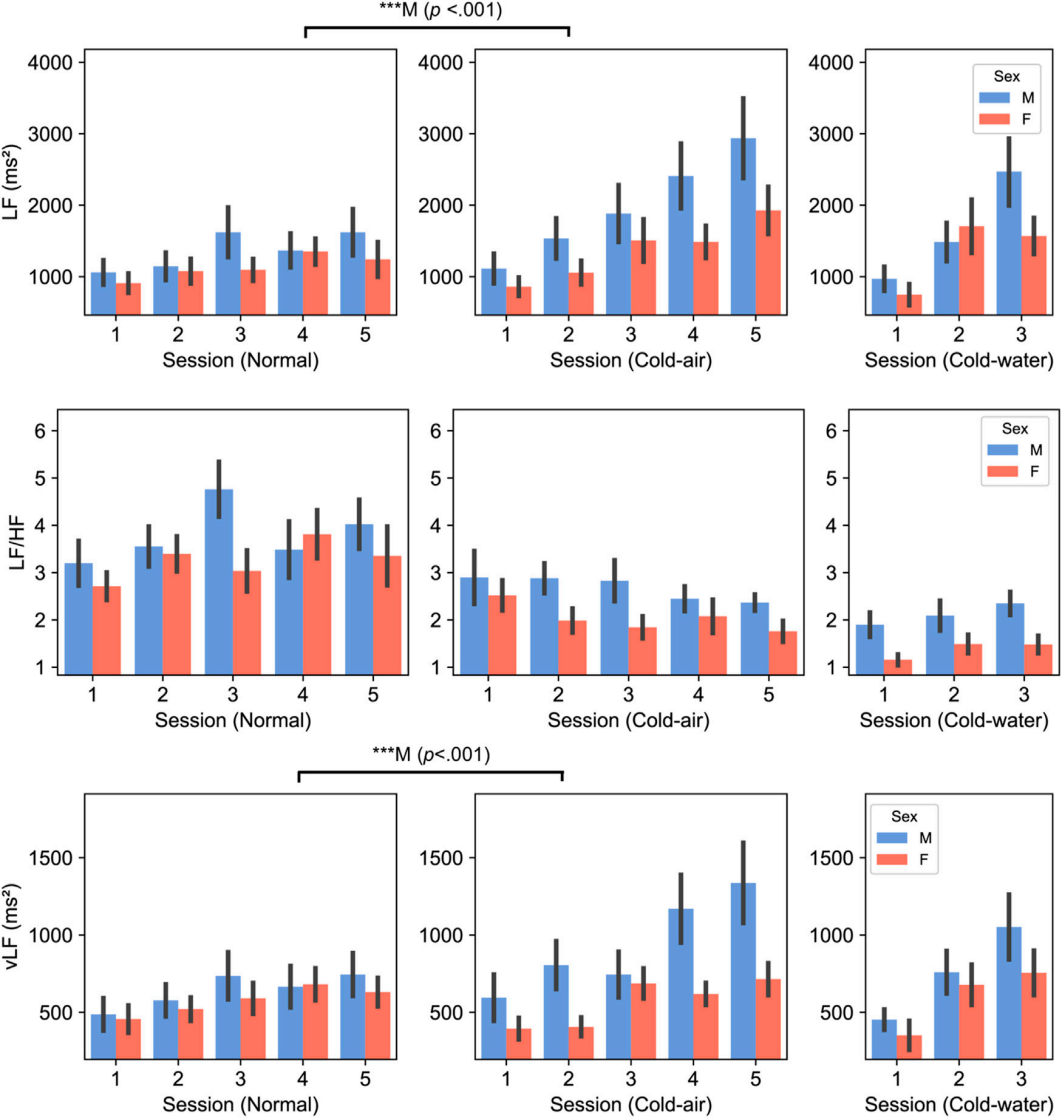
LF (top panel), LF/HF (middle panel), and vLF (bottom panel) between male and female subjects. *P*-values displayed at the top of the figure indicate significant differences between the normal and cold-air conditions for each sex.

SDNN was significantly higher in the cold-air condition than in the normal condition for both males and females (five-session, *p* < .01) (Supplementary Figure S5). LF/HF, LF, and vLF showed different patterns between males and females (Figure 4). LF/HF showed higher values in males compared to females [three-session analysis, *F* (1,38) = 5.370, *p* = .026]. Additionally, male subjects showed significantly higher LF and vLF in the cold-air condition compared to the normal condition (*p* < .001). Time-domain HRV indices (RMSSD and SDNN) and HR did not show statistical difference between males and females.

## Discussion

We studied the differences between male and female subjects in their cognitive performance and autonomic functions during exposure to three environments, including room-temperature-air/normal, cold-air, and cold-water conditions. Twenty-two male and nineteen female subjects performed a battery of cognitive tasks in these conditions, while HRV and EDA data were collected passively. In our study, subjects were exposed to three different environmental conditions that were pseudo-randomly assigned, based on the availability of the experiential units. However, the comparison between the expected (i.e., uniform) frequency of each permutation of the three conditions and our pseudo-random frequency was not statistically significant (Chi-square, *p* = .193). In future studies, true randomization should be considered to minimize potential bias.

Regarding cognitive performance, the reaction times of SPD, PRO, and CDS tasks showed differences between normal and cold-air conditions for either or both sexes, while none of the error-related indices did. Interestingly, in the cold-air condition compared to the normal condition, male subjects showed significantly improved SPD and CDS performance while female subjects exhibited significantly deteriorated SPD, PRO, and CDS performance. The reaction time of PRO in female subjects was significantly higher in the first session (i.e., around 10 min of exposures) of cold-air condition compared to all sessions in the normal condition. This may suggest that cold-air exposure female subjects showed deteriorated performance in the beginning period of cold-air exposures, but the combination of learning effect and habituation in the cold-air condition mitigates the affected performance. The reaction time of CDS also showed significantly deteriorated performance for female subjects in the cold-air condition compared to the normal condition. These performance decrements in females are possibly due to females typically feeling less comfortable than males in cold environments (Kaikaew et al., 2018; Maykot et al., 2018). Alternatively, it may be due to a result of cooling of the extremities of the body. Studies showed that females generally experience lower hand blood flows when exposed to cold attributed by increased vascular reactivity (Daanen, 2003). For example, pioneers of this field revealed that finger temperature was significantly warmer in males compared to females during exposure to 0°C cold air for 0–40 min, followed by stabilization between 40 and 75 min of exposures with no significant difference between males and females (Reading et al., 1997). Despite showing decreased performance due to cold environments, female subjects showed faster reaction time than male subjects in memory and attention tasks (i.e., CDS) for the normal and cold-water conditions. Our results are in agreement with other studies which have demonstrated that females outperform males across various short-term memory tasks (Kaushanskaya et al., 2011; Gabriel and Sridevi, 2016; Adyalkar, 2019; Theofilidis et al., 2020). Our findings provide a novel perspective that cold-air exposure may exert an influence on this sex-based disparity.

Our EDA analysis also revealed sex differences in response to cold-air exposures and cold-water immersion. Both male and female subjects showed significantly lower PhEDA and TVSymp in the cold-air and cold-water conditions compared to the normal condition, while only female subjects showed significantly lower values in the cold-water condition than in the cold-air condition. Both male and female subjects showed significantly higher TonEDA in the normal condition compared to the other conditions, while only male subjects showed significantly decreased values in the cold condition compared to the normal condition. The decreased TonEDA in female subjects was marginally significant in the cold air condition compared to the normal condition (*p* = 0.062). In summary, both males and females showed generally lower values in EDA during the cold-air and cold-water conditions compared to the normal condition, and lower values in the cold-water condition compared to the cold-air conditions. Most EDA indices did not show a significant difference between males and females for any conditions. Bari also found no significant difference in both PhEDA and TonEDA from subjects who underwent sympathetic stimuli (Bari, 2020). Our findings provide further evidence that vasoconstriction in the skin results in decreased openings of sweat glands, subsequently leading to decreased skin conductivity (Edelberg, 1964; Deltombe et al., 1998). Interestingly, TVSymp during the normal condition was significantly higher in male subjects than in female subjects, which has shown higher sensitivity in response to sympathetic stimuli compared to other EDA indices including PhEDA and TonEDA (Posada-Quintero et al., 2016; Kong et al., 2021c). This higher degree of sensitivity capable of differentiating SNS activation between males and females, possibly due to the cognitive tasks, may be diminished by the cold environment.

For HRV analysis, we found differences in the LF/HF ratio, LF, and vLF indices. LF/HF in male subjects was significantly higher than in female subjects across all conditions, which is consistent with other studies (Koenig and Thayer, 2016; Ramalho et al., 2017). This generally indicates a relatively greater cardiac sympathetic tone or reduced cardiac parasympathetic tone in males. Regarding LF and vLF, male subjects showed significant increases in LF and vLF in the cold-air condition compared to the normal condition, while female subjects did not exhibit any differences. Mäkinen et al. also showed significant increases of LF in ten male subjects who underwent cold (10°C) environment exposure for 2 h (Mäkinen et al., 2008). Additionally, Lundell et al. (2020) also showed increased LF and vLF in four male Navy divers who underwent 0°C cold-water dives (Lundell et al., 2020). While we did not find significant effects in female subjects, Matsumoto et al. found a significant increase in LF and vLF in twelve female subjects who underwent control (25°C) or cold-air exposure (10°C) conditions for 15 min (Matsumoto et al., 1999). Interestingly, they reported no significant increase in LF and vLF for another 12 subjects in an obese group. This may be because our group’s BMI is closer to the obese group than the non-obese group (Table 4). Several studies have shown that obesity is associated with reduced SNS activity, which is inversely correlated with reduced brown adipose tissue activity, contributing to impaired thermogenesis in response to cold exposure (Jung et al., 1979; Himms-Hagen, 1989; Nielsen et al., 1993; Matsumoto et al., 1999). It is thought that vLF is closely connected to the thermoregulation mechanism (Fleisher et al., 1996; Thayer et al., 1997). The vLF feature may be a key indicator of the fact that only female subjects showed impaired cold-stressed cognitive performance in our analysis.

**TABLE 4:** BMI and Fisher’s Ratio (Ciortan, 2019) of obese and non-obese groups in (Matsumoto et al., 1999).

Female subjects	BMI	Fisher’s Ratio with our group
Obese group (n = 12)	26.3 ± 0.74	0.831
Non-obese group (n = 12)	18.5 ± 0.18	1.153
Our group (n = 19)	23.0 ± 3.9	0

In addition to our observations of differences in autonomic mechanisms, there are other factors that can influence sex differences in respond to cold exposures. Males tend to have more lean mass, while females tend to have more fat mass in comparison (Bredella, 2017). Our results also showed significantly higher fat percentage in female subjects compared to male subjects. Additionally, males generally accumulate adipose tissue around the trunk and abdomen, whereas females typically accumulate tissue around the hips and thighs (Bredella, 2017), which was shown in our demographic information with thicker thigh skinfolds in female subjects. This difference in adipose tissue distribution may lead to variation in thermoregulation between males and females, as studies found different regional distributions of brown adipose tissue (Heaton, 1972; Leitner et al., 2017). Furthermore, females have a higher proportion of estrogen than males, which can affect their response to cold temperatures (Charkoudian and Stachenfeld, 2016; Herz et al., 2021). For example, increased estrogen augments vasodilation by directly affecting peripheral blood vessels, which increases blood flow to the skin and thus helps maintain warmer skin temperatures in cold environments (Charkoudian and Stachenfeld, 2016). Moreover, some studies showed that estrogen can influence memory and spatial performance (Patel et al., 2022; Joue et al., 2024). In this study, however, we did not consider these factors, including effect of hormones. These should be considered, along with menstruation cycle, in future studies.

We demonstrated that sex differences play an impactful role in autonomic functions and cognitive performance during cold-air exposures and cold-water immersion. However, there was no significant effect of sex and environmental conditions on short-term exposure time, except for one reaction time task. One of our findings is that females may require habituation time (around 30 min) in cold environments for tasks that require fast reaction time, or sufficient training for the task. Another finding is that males may have better reaction time performance in cold-air exposure than in normal conditions. Therefore, different environmental strategies should be provided depending on sex, to achieve optimized performance in reaction time, such as thermal clothing to mitigate different environmental temperatures. Additionally, sex should be considered as one of the main factors when analyzing cognitive performance obtained during cold environment. Individual variations are also important to consider, as response to cold can vary widely depending on a person’s body composition, genetics, and other factors.

### Limitations

One limitation of this study is the absence of blood pressure measurements. Blood pressure can provide insights into the effects on arterial and central venous pressure, such as vasoconstriction and hydrostatic pressure. Additionally, we did not account for the potential of baroreflex resetting under different conditions. For instance, a previous study demonstrated that cold exposure can affect baroreflex sensitivity and overall vagal activity (Pikkarainen et al., 2023). Our study did not consider the potential impact of different temperature conditions on baroreflex resetting, which may have affected HRV indices. These should be investigated with blood pressure measurements in future studies. Another limitation is complexity of EDA analysis associated with cold exposure. The increase in skin sympathetic nerve activity during cold exposure is thought to be primarily noradrenergic (Charkoudian, 2010), while EDA is primarily mediated by acetylcholine (Posada-Quintero and Chon, 2020). Muscarinic receptors, which respond to acetylcholine, are mainly associated with the PNS but also play a significant role in stimulating the sweat glands under the control of the SNS (Kudlak and Tadi, 2023). A rat study exhibited decreased acetylcholine responding to short-term cold stress, followed by gradual recovery (Stillman et al., 1997). Similarly, our tonic EDA index showed an increasing trend for both males and females over time in cold-air conditions (Figure 3; Supplementary Table S4). The decreasing trend in Phasic EDA, NSSCR, and TVSymp may be due in a larger part to subjects becoming accustomed to the cold stress and the series of cognitive tasks. (Figure 3; Supplementary Table S4). Interestingly, cold-water condition showed decreased tonic EDA over time, while other metrics increased for males but decreased for females (Figure 3; Supplementary Table S5). This suggests that there may be sex difference in hydrostatic effect to EDA. However, this should be investigated further, as no significant combined effect of exposure time, conditions, and sex was observed in the EDA indices (except for TVSymp in the normal condition). Thus, EDA measured during the early phase of cold exposure may need to have additional considerations made during analysis compared to EDA measured at normal temperatures. EDA might have been affected by the direct impact of cooling on the skin, even though a study showed no correlation between the degree of cooling and the time of onset or intensity of sweating (Glaser and Lee, 1953). However, their study is limited in assessing the effect on the phasic component of EDA and also tested only males. These should be further investigated in future studies. We did not account for the effect of smoking (despite no significant difference between males and females, Chi-square test, *p* = .284), hydration (at least a cup of water for the core temperature pill), shivering during cold exposures, and thermal perception and comfort. The effects of these factors should be investigated in future studies.

Another significant limitation of this study is the lack of investigation into the menstrual cycle. The menstrual cycle, an integral component of the female reproductive system, involves regular cyclic changes that prepare the body for pregnancy and fertilization, regulated by hormones such as follicle-stimulating hormone, luteinizing hormone, estrogen, and progesterone (Thiyagarajan et al., 2022). One notable physiological effect associated with hormonal fluctuations during the menstrual cycle is the alteration in thermoregulatory responses, with an increased core body temperature during the luteal phase compared to the follicular phase (Baker et al., 2020). Specifically, females in the luteal phase exposed to rapid cold air have a constantly higher core body temperature compared to those in the follicular phase, along with a higher threshold for shivering, sweating, and cutaneous vasodilation (Hessemer and Bruck, 1985; Charkoudian and Johnson, 1999; Baker et al., 2020). Moreover, the menstrual cycle influences cognitive function (Farage et al., 2008; Baker et al., 2020). For instance, researchers have found that performance on simple reaction tasks during the luteal phase is better when core body temperature is higher (Baker et al., 2020). Another notable pattern is that performance on mental rotation tests (spatial ability) peaks during menstruation but is lowest during the mid-luteal phase (Farage et al., 2008). Furthermore, the menstrual cycle affects both EDA (Gómez-Amor et al., 1990) and HRV (Brar et al., 2015). Gómez-Amor et al. showed that females in the ovulatory phase exhibited significantly greater values in both SCR and SCL to auditory stimuli, than did those in menstrual, luteal, and premenstrual phases (Gómez-Amor et al., 1990). For HRV, Brar et al. demonstrated that HRV indices, including LF/HF, LF, and vLF, which showed sex differences in our analysis, were statistically significant across the menstrual cycle (Brar et al., 2015). In their study, LF, vLF, and LF/HF ratio were higher during the luteal phase compared to the follicular phase. As the menstrual cycle can influence thermoregulatory responses, cognitive and autonomic functions, future studies should include female participants across various stages of the menstrual cycle.

## Data Availability

The raw data supporting the conclusions of this article will be made available by the authors, without undue reservation.

## Author disclaimer

The views expressed in this article reflect the results of research conducted by the authors and do not necessarily reflect the official policy or position of the Department of the Navy, Department of Defense, nor the United States Government. The study protocol was approved by the Institutional Review Boards at the University of Connecticut (UConn) and the Naval Submarine Medical Research Laboratory (NSMRL), in compliance with all applicable Federal regulations governing the protection of human subjects (protocol #NSMRL.2022.0002). One of the authors is an employee of the U.S. Government. This work was prepared as part of their official duties. Title 17, U.S.C., §105 provides that copyright protection under this title is not available for any work of the U.S. Government. Title 17, U.S.C., §101 defines a U.S. Government work as a work prepared by a military Service member or employee of the U.S. Government as part of that person’s official duties. Regarding the use of ScottCare, Cardiac Direct, Equivital, Empatica, Inquisit, and similar products in this article: reference to any specific commercial product, process or service or the use of any trade, firm or corporation name is for the information and convenience of the public, and does not constitute endorsement, recommendation, or favoring by the Department of the Navy.
