# Supplementary material for: Sex differences in autonomic functions and cognitive performance during cold-air exposure and cold-water partial immersion
Source: Front Physiol. 2024 Oct 16;15:1463784. doi: 10.3389/fphys.2024.1463784 (PMC11521960; doi:10.3389/fphys.2024.1463784)
Supplement: Supplementary file 1 [file DataSheet1.pdf]

Screening Questionnaire

1. Do you have a pacemaker or any other implanted electro-medical device?
2. Have you been diagnosed with arrhythmia or any other forms of irregular heartbeat?
3. Have you ever taken prescription medication for blood pressure or for a heart condition?
4. Do you have a disordered or impaired gag reflex?
5. Do you have difficulties swallowing?
6. Have you had issues with esophageal motility (trouble with contractions in your esophagus)?
7. Have you been diagnosed with any gastrointestinal disorders, diverticulitis (inflammatory or infected digestive tract), or obstructive gastrointestinal tract disease?
8. Have you had gastrointestinal surgery in the past?
9. Have you been diagnosed with ileus or any other motility (movement) disorders of the gastrointestinal tract?
10. Are you scheduled or do you plan to undergo magnetic resonance imaging (MRI) or nuclear magnetic resonance (NMR) scans within 5 days of an experimental trail day?
11. Are you scheduled or do you plan to board a commercial aircraft within 5 days of an experimental trial day?
12. Are you or could you be pregnant at this time?
13. Do you have any skin conditions that could be exacerbated by or react to exposure to cold environments?
14. Do you have any skin conditions that could be exacerbated by or react to adhesive material (e.g., allergies, edema, sensitivities, etc.)?

Children; pregnant women, fetuses, or, neonates; or prisoners are **NOT** involved.

Table S1. Available segments between male and female subjects.

Sites	8-point weights (ISO 9886:2004)		3-point weights (Our study)
Forehead	0.07	× 3.125	0.21875
Right scapula	0.175		
Left upper chest	0.175		
Right arm in upper location	0.07		
Left arm in lower location	0.07		
Left hand	0.05	× 3.125	0.15625
Right anterior thigh	0.19		
Left calf	0.2	× 3.125	0.625
Summation	1.00		1.00

Table S2. Available segments between male and female subjects.

		Normal		Cold-air		Cold-water		Total		Number of Subject	
		M	F	M	F	M	F	M	F	M	F
Total Segments		105	95	105	95	66	57	276	247	22	19
Temperature		61	46	60	58	18	38	139	142	20	19
HRV		90*	87	88	88	75	70	253	245	21	19
EDA		105	95	105	95	66	57	276	247	22	19
TASK Battery	GNG	105	95	105	95	66	57	276	247	22	19
	SPD	85	85	85	85	54	51	224	221	17	17
	PRO	105	95	105	95	66	57	276	247	22	19
	SRT	105	95	105	95	66	57	276	247	22	19
	CDS	55	80	55	80	36	48	146	208	12	16

* Four segments are from PPG, and the others are from ECG.

Table S3. BMI Category table from the WHO's BMI guideline.

	Male	Female	BMI (kg/m^2)
Underweight (Moderate thinness)	0	0	16.0 – 16.9
Underweight (Mild thinness)	0	1	17.0 – 18.4
Normal	12	15	18.5 – 24.9
Overweight (Pre-obese)	9	2	25.0 – 29.9
Obese (Class I)	1	0	30.0 – 34.9
Obese (Class II)	0	1	35.0 – 39.9
Obese (Class III)	0	0	≥ 40.0

WHO: World Health Organization

Table S4. Rate of increase/decrease in mean of each EDA index between sessions in the cold-air condition.

Session	Phasic EDA		Tonic EDA		TVSymp		NSSCR 0.05		NSSCR 0.01		NSSCR 0.005	
	M	F	M	F	M	F	M	F	M	F	M	F
2	-22.5%	-21.4%	14.8%	15.8%	-53.5%	-56.8%	-37.1%	-35.2%	-28.4%	-35.5%	-21.1%	-30.0%
3	-52.3%	-25.9%	23.3%	19.5%	-60.2%	-69.2%	-50.5%	-54.2%	-40.5%	-37.6%	-32.9%	-33.3%
4	-45.5%	-73.0%	34.8%	25.0%	-55.3%	-81.7%	-37.1%	-62.5%	-33.3%	-50.8%	-26.4%	-45.7%
5	-66.2%	-69.5%	47.7%	40.0%	-59.2%	-76.7%	-44.0%	-46.1%	-34.6%	-38.3%	-25.9%	-34.4%

$$Rate\ of\ increase/decrease\ (\%) = 100 \times \frac{EDA_{current\ session} - EDA_{session1}}{EDA_{session1}}.$$

Table S5. Rate of increase/decrease in mean of each EDA index between sessions in the cold-water condition.

Session	Phasic EDA		Tonic EDA		TVSymp		NSSCR 0.05		NSSCR 0.01		NSSCR 0.005	
	M	F	M	F	M	F	M	F	M	F	M	F
2	71.6%	-67.7%	-21.6%	-22.7%	32.9%	-18.2%	100.0%	-33.3%	11.9%	-15.8%	-23.3%	-31.1%
3	94.9%	-78.1%	-26.0%	-20.3%	40.8%	-24.1%	258.8%	-55.6%	53.2%	-5.3%	3.0%	-15.5%

$$Rate\ of\ increase/decrease\ (\%) = 100 \times \frac{EDA_{current\ session} - EDA_{session1}}{EDA_{session1}}.$$

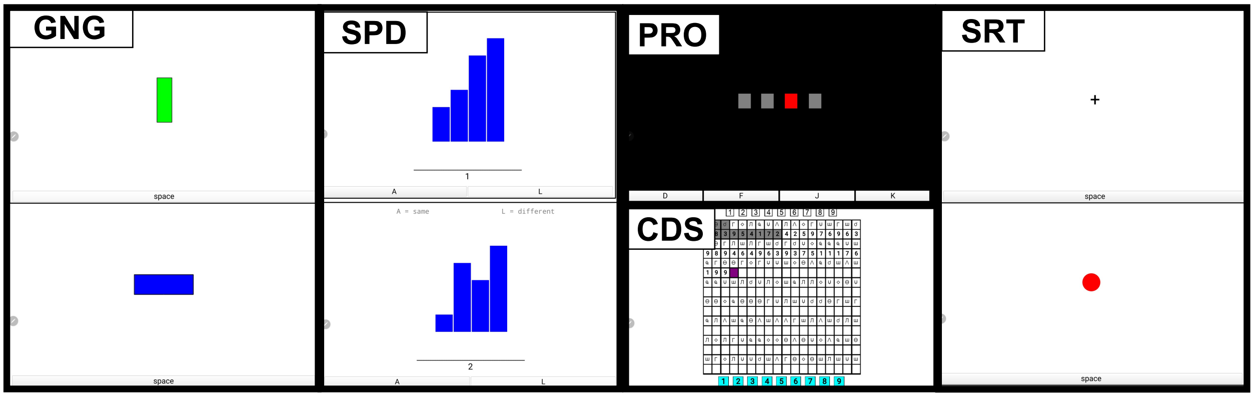

Figure S1. An example of the task battery.

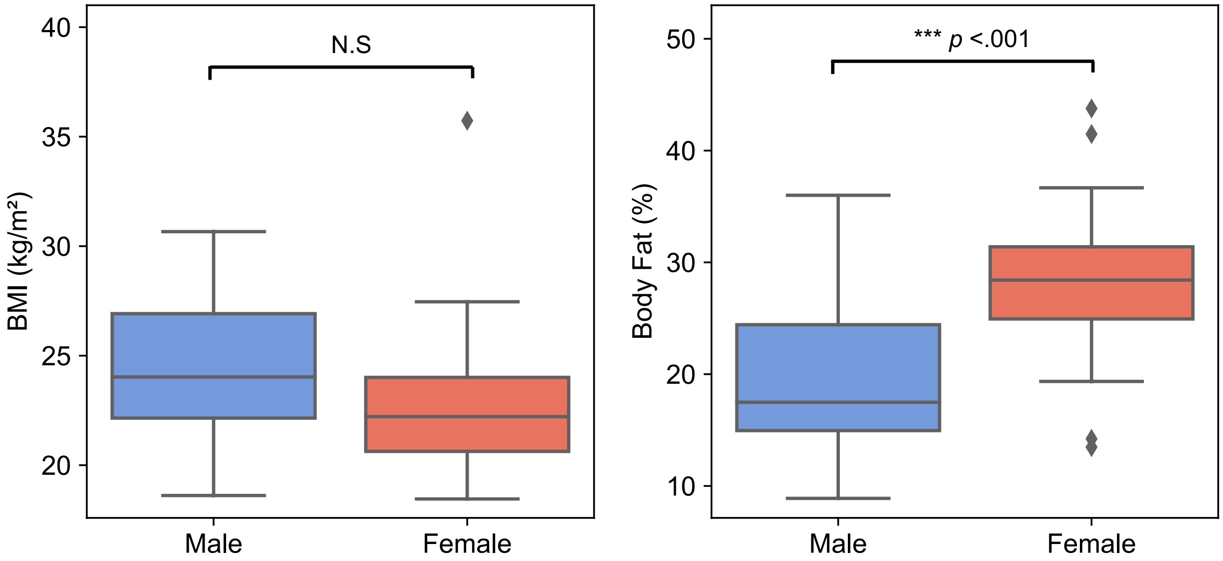

Figure S2. BMI (**left panel**) and Body Fat (**right panel**) between male and female subjects. Out of the 22 male subjects, 12 were considered normal weight, 9 were overweight, 1 was classified as obese class I, and 1 was categorized as obese class II, according to the World Health Organization (WHO)'s BMI guideline (A healthy lifestyle - WHO recommendations, n.d.). For the 19 female subjects, 15 were of normal weight, 2 were overweight, 1 was underweight, and 1 was obese class II (Table 3). The median body fat for males and females was 17.5% and 28.4%, respectively.

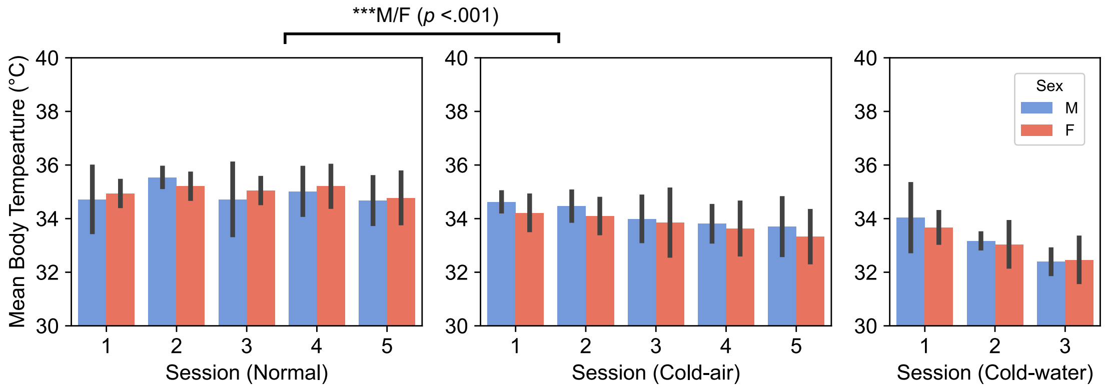

Figure S3. Mean Body Temperature (MBT) between males and females (Mean±SEM). P-values displayed at the top of the figure indicate significant differences between the normal and cold-air conditions for each sex. Sex did not have a significant effect on MBT. There was significant effect of combined factors of sex and condition in the five-session ($F(1,186) = 4.180$, p=.042). However, post hoc comparisons revealed no statistically significant differences between the sexes for each condition.

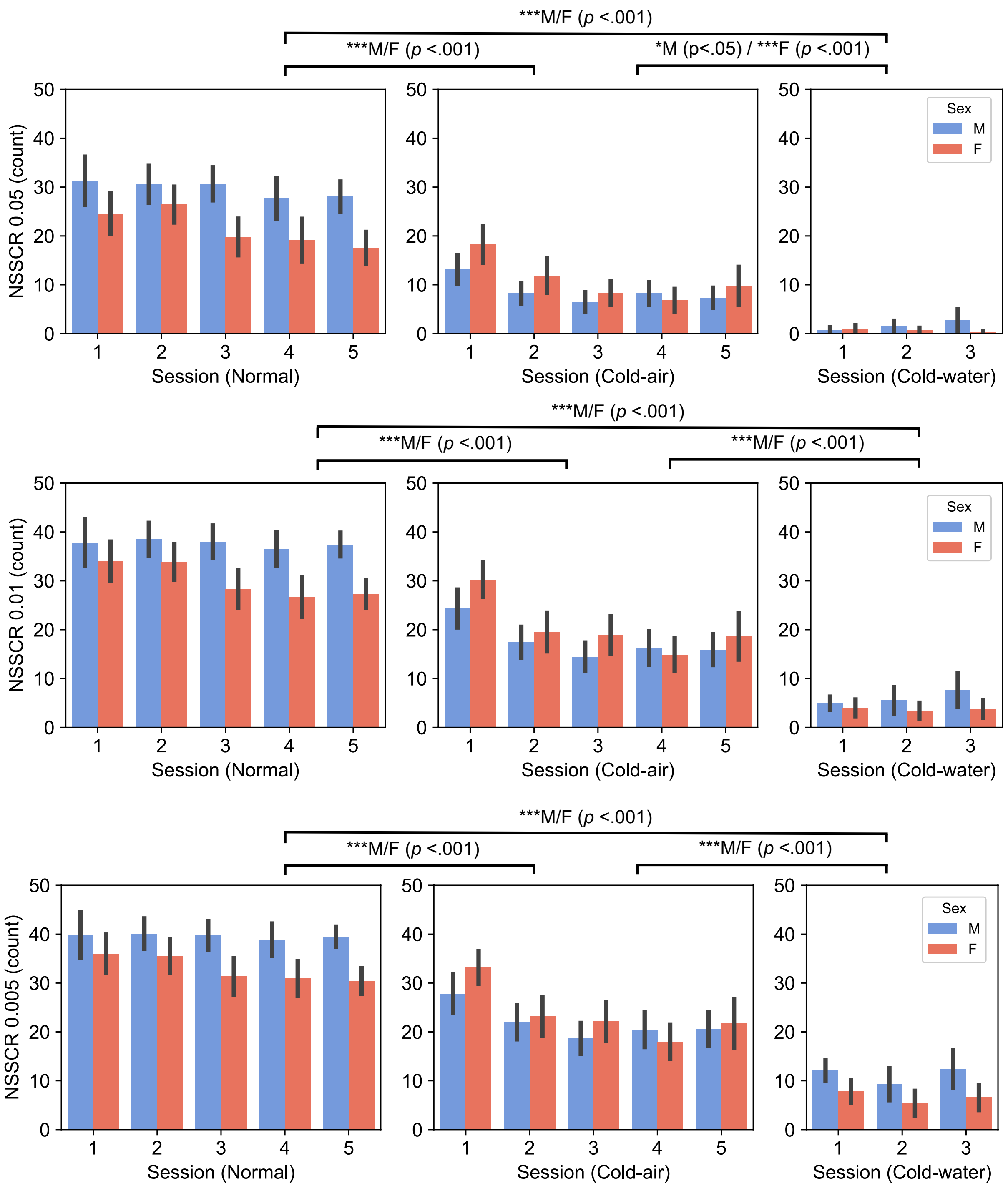

Figure S4. NSSCR 0.05 (**top panel**), NSSCR 0.01 (**middle panel**), and NSSCR0.005 (**bottom panel**) between male and female subjects. P-values displayed at the top of the figures indicate significant differences between the normal and cold-air conditions for each sex.

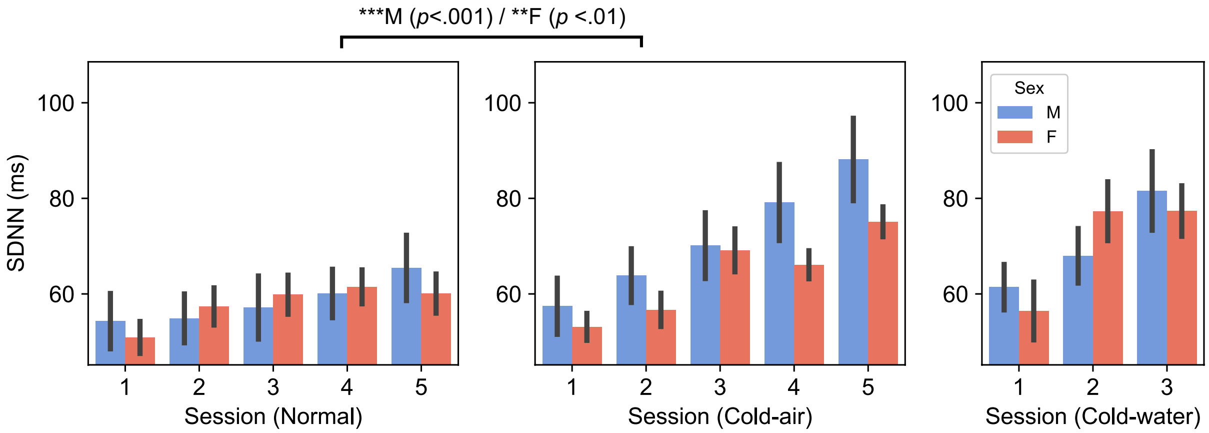

Figure S5. SDNN between male and female subjects. P-values displayed at the top of the figure indicate significant differences between the normal and cold-air conditions for each sex. Both male and female subjects showed significantly higher SDNN values in the cold-air condition than in the normal condition (five-session, $p < .01$).
